# Intraoperative anaphylaxis due to gelofusine in a patient undergoing intramedullary nailing of the femur: a case report

**DOI:** 10.1186/1757-1626-2-12

**Published:** 2009-01-06

**Authors:** Ioannis Polyzois, Andrina Lampard, Paras Mohanlal, Evgenios Tsiridis, Nikolaos Manidakis, Eleftherios Tsiridis

**Affiliations:** 1 Academic Orthopaedic Unit, Leeds General Infirmary, Leeds School of Medicine, Leeds, LS1 3EX, UK; 2 Blue Cross Hospital, Cardio-Thoracic Anaesthetic Unit, Thessaloniki, Greece

## Abstract

**Background:**

Although uncommon, anaphylaxis due to a colloid plasma expander can occur peri-operatively

**Case presentation:**

We present a case of an intra-operative cardiac arrest in a 72 year old Caucasian male patient who underwent prophylactic intramedullary nailing for a proximal femoral metastasis from prostate cancer. The patient was resuscitated successfully and the procedure was completed uneventfully. Elevated serum tryptase levels confirmed the diagnosis of an anaphylactic reaction and positive allergy skin prick testing identified gelofusine as the causative agent.

**Conclusion:**

A high index of suspicion, prompt diagnosis and rapid institution of treatment are essential for a safe outcome following such reactions. To our knowledge, this is the first published report of such a severe reaction to gelofusine infusion that occurs during an orthopaedic procedure.

## Background

Colloid plasma expanders are widely used during surgery and play a key role in resuscitation of the severely hypovolaemic patient [1]. They provide intravascular volume expansion and help reduce transfusion requirements whilst also allowing time for full blood cross matching to be carried out. Anaphylactoid reaction to Gelofusine, that contains succinylated gelatin and other plasma expanders carries an estimated incidence of 0.07–0.15% [1,2]. However, this can prove life-threatening if not promptly recognized and accordingly treated.

These reactions are normally type I, IgE-mediated and cause production of antibodies through prior sensitization, although in many cases they may occur without any previous documented exposure. The reaction is termed anaphylactoid when there is no known prior exposure for the production of the antibody-antigen reaction of true anaphylaxis [1,3].

## Case presentation

**Figure 1:**
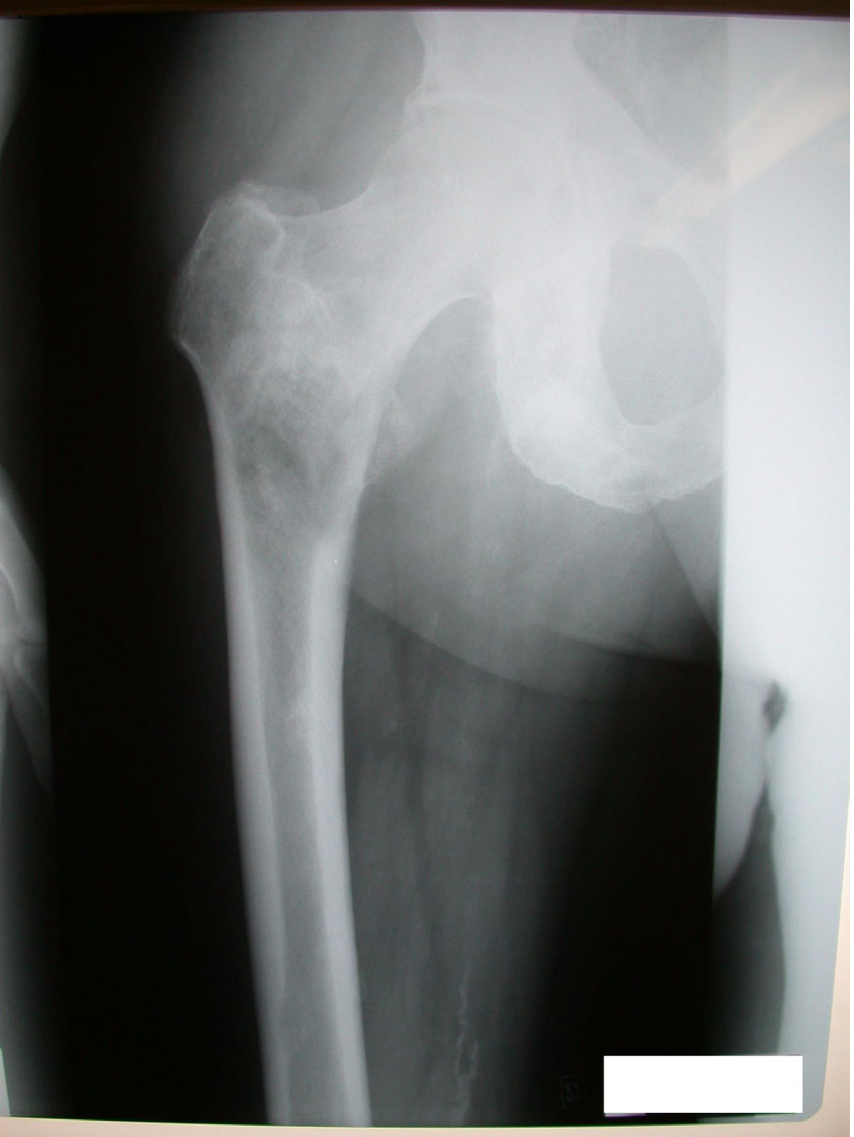
**Anteroposterior (AP) view radiograph of the left femur demonstrating the large lytic metastasis in the subtronchanteric area**.

**Figure 2:**
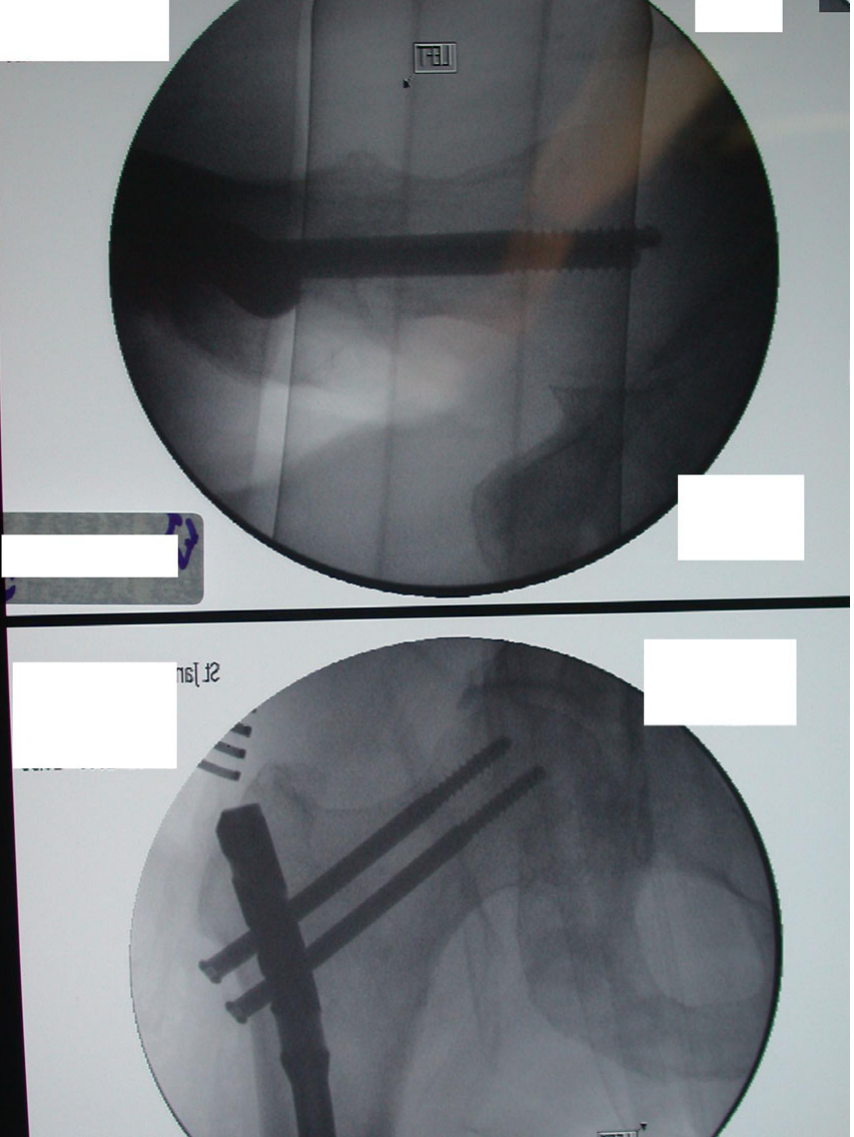
**AP and lateral image intensifier views showing optimal positioning of the proximal locking screws and the intramedullary nail in the left proximal femur**.

**Figure 3:**
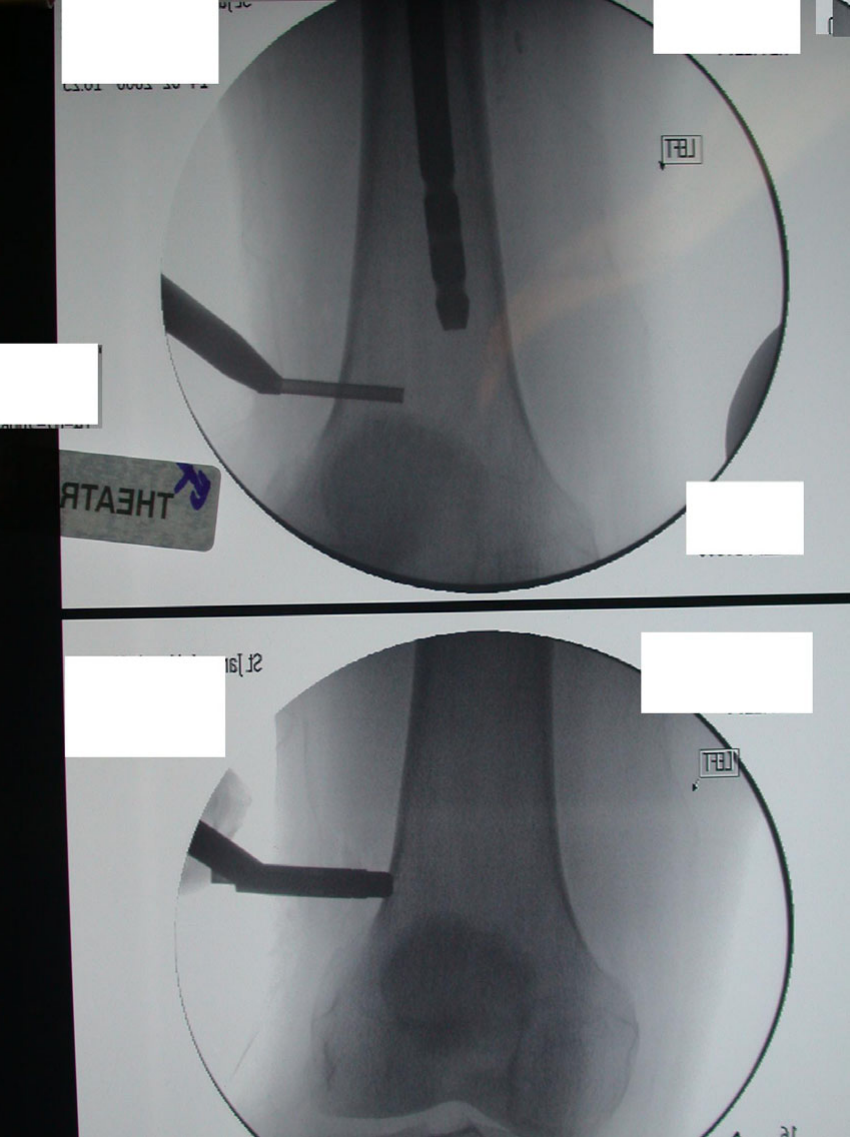
**AP views of the left distal femur demonstrating drilling and creation of a distal venting hole**.

A 72 year old male Caucasian patient was referred to our unit with a metastatic bony deposit involving his left proximal femur from a known primary prostatic carcinoma. Commonly, prostatic metastases are sclerotic in nature. However detailed radiological evaluation demonstrated a large lytic lesion in this case (Figure 1). Due to the high risk of an impending pathological fracture, the decision was taken to perform prophylactic intramedullary nailing of the left femur (Figure 2). In order to reduce the increased intramedullary pressure during nail insertion, venting of the distal end of the femur was performed (Figure 3).

The patient did not have any other significant past medical history and he was not known to be allergic to any medication. He had surgical procedures for other conditions in the past requiring general anaesthesia and had an uneventful recovery in every case.

During the procedure, the patient suffered a cardiac arrest. Fat embolism was initially thought to be the cause of this event due to the increased intramedullary pressure during reaming and nail insertion. Following successful resuscitation, a marked generalized erythematous rash was noticed which lasted for about thirty minutes. This raised the concern of a possible hypersensitivity reaction to one of the anaesthetic agents. Our patient received in total eight different drugs namely propofol, atracurium, morphine, ondansentron, dexamethasone, gelofusine, paracetamol and diclofenac. Interestingly, it was noted that the cardiac arrest occurred following a dose of atracurium and an infusion of gelofusine. Consequently, it was thought that either one of those two agents could have been responsible for the anaphylactic response.

Serum mast cell tryptase levels taken one hour following the event, demonstrated a level of 190 ng/ml, which dropped to 60 ng/ml upon repeating the levels twelve hours later (normal range 3–23 ng/ml). The raised levels of mast cell tryptase were consistent with mast cell degranulation. The latter strongly suggested a hypersensitivity reaction. Furthermore, allergy skin prick tests were performed to all the drugs used during the procedure. Interestingly, they showed a positive reaction to gelofusine only, confirming it as the causative agent.

## Discussion

Plasma expanders play an important role in trauma resuscitation as well as during the peri-operative period. However there is controversy regarding the relative merits of colloid versus crystalloid solutions as colloids can induce an anaphylactoid reaction which could potentially prove life threatening if left untreated [4].

Such reactions are more common in male patients with atopy and often occur within ten minutes of commencing the infusion, hence the need for early and frequent monitoring [1]. The reactions are graded in severity on a scale of I-V and tend to be under-reported [2]. Nevertheless, with the increasing use of these agents there are sporadic reports of such adverse reactions in the literature [3,5-7].

In a large multi-centre prospective trial conducted by Ring and Messner which involved 200,906 infusions of colloid substitutes, sixty-nine cases of anaphylactoid reactions were observed. Specifically, the incidence of severe reactions including shock, cardiac and/or respiratory arrest was found to be 0.003% for plasma protein solutions, 0.006% for hydroxylethylstarch, 0.008% for dextran and 0.038% for gelatin solutions [2].

Not all gelatins share the same molecular structure; Haemaccel and Plasmagel are urea-linked, whilst Gelofusine is a succinate-linked gelatin. Hepner and Castells stated that there is no known cross-reactivity between different colloids, so a particular allergy to one should not preclude the use of another [8]. Whilst this is probably true of colloids of highly differing chemical composition, it is not the case between Haemaccel and Gelofusine which differ only in their linkage to urea or succinate. Cross-reactivity between these two colloids has been documented by intradermal skin prick testing [9]. Consequently, any patient known to be allergic to one should be assumed as being allergic to the other until proven otherwise.

There have been case reports of anaphylactoid reactions to all of the gelatin-based colloids, with varying degrees of response severity. In 1979 Freeman reported a case of severe anaphylaxis to Haemaccel which unfortunately resulted in death. Autopsy findings of mucus in the bronchioles, indicating severe anaphylactic bronchospasm were attributed to reaction to the infused colloid, though no concrete evidence of the role of Haemaccel was found [6]. Often, due to polypharmacy during anaesthesia it is difficult to elucidate a single agent causing the anaphylactic response. In severe reactions it may become necessary to abandon the operative procedure until the patient has been resuscitated and conduct further investigations to identify the cause [5]. Great care must be taken once there is a suspicion of allergy due to the possibility of reaction to other gelatin-based colloids or escalating anaphylactic response with further infusion [7]. This scenario was exemplified by Vervloet et al, who described three cases of anaphylaxis due to modified fluid gelatin Plasmagel. One of these occurred during an operative procedure which had to be abandoned and in one patient a repeat infusion of Plasmagel caused anaphylactic shock [5].

Diagnosis can be a challenge in such circumstances. Some features of anaphylactic response to an agent are similar to the effects of anaesthesia itself. Most anaesthetic agents can cause vasodilation, hypotension and potential cardiopulmonary dysfunction due to their direct and indirect effects on the cardiovascular system, and distinguishing this from an anaphylactic reaction can prove difficult. In our case, the cardiorespiratory arrest could have been due to a number of different causes, including fat or pulmonary embolus. It was only after careful observation that the developing erythematous rash was noted and the possibility of anaphylaxis considered.

Elevated serum tryptase levels are indicative of mast cell degranulation and are very helpful in the differential diagnosis of anaphylaxis. Concentrations peak one hour after the hypersensitivity reaction and can usually last for several hours thereafter. Comparison with the baseline values, taken a few weeks after the event, may confirm or exclude the diagnosis. However this does not define the causative agent. The standard diagnostic technique for this is skin prick testing. This may be complemented by detection of specific IgE by radioimmunoassay [10].

Apostolou et al have reported the use of *in vitro *basophil activation test (BAT) as a safe and reliable assay test to detect gelofusine sensitivity. This method uses detection of surface expression of lysosomal membrane glycoprotein CD63 on activated basophils [11]. The leucocyte histamine release test (LHR) which can measure histamine release in response to gelatin solutions *in vitro *has been described but remains mostly a research tool [5].

## Conclusion

Even though colloid plasma expanders carry a risk of anaphylactoid reaction, this is small when compared to common drugs such as penicillin, which carries a risk of adverse reaction from 1–5% [12]. Plasma expanders provide intravascular volume expansion and help reduce transfusion requirements. However, the use of these agents should be done with caution. A high index of suspicion and a prompt diagnosis should ensure successful resuscitation in the event of anaphylaxis.

## Consent

Written informed consent was obtained from the patient for publication of this case report and accompanying images in the Cases Journal.

## Competing interests

The authors declare that they have no competing interests.

## Authors' contributions

IP conducted a literature search and prepared the final manuscript. AL conducted a literature search and contributed to preparation of the manuscript. PM prepared the first draft of the manuscript. ET conducted a literature search and contributed to preparation of the final manuscript. NM contributed to the preparation of the final manuscript. ET supervised the manuscript and treated the patient. All authors contributed equally in collecting patient data and editing radiographic images.
